# Effect of different dietary energy/protein ratios on growth performance, reproductive performance of breeding pigeons and slaughter performance, meat quality of squabs in summer

**DOI:** 10.1016/j.psj.2023.102577

**Published:** 2023-02-10

**Authors:** Jie Peng, Weiying Huang, Wei Zhang, Yanlin Zhang, Menglin Yang, Shiqi Zheng, Yantao Lv, Hongyan Gao, Wei Wang, Jian Peng, Yanhua Huang

**Affiliations:** ⁎ Innovative Institute of Animal Healthy Breeding, College of Animal Science and Technology, Zhongkai University of Agriculture and Engineering, Guangzhou, Guangdong 510225, China; † College of Animal Science and Technology and Animal Medicine, Huazhong Agricultural University, Wuchang, Wuhan 430000, China; ‡ Guangdong Laboratory for Lingnan Modern Agricultural, Guangzhou 510642, China

**Keywords:** dietary energy/protein ratio, growth performance, reproductive performance, slaughter performance, meat quality

## Abstract

Large-scale pigeon farming in China is gradually increasing. However, studies on the basic nutritional requirements of breeding pigeons during lactation, which greatly influence the productivity and economic benefits of pigeon breeding, remain scanty. The objective of this study was to determine the optimal dietary energy/protein ratio requirements for lactating pigeons in summer. A total of 576 pairs of Mimas breeding pigeons were randomly divided into 12 groups (*n* = 48 per treatment), and each pair bred 4 squabs. A two-way ANOVA design with different protein levels (15%, 16%, 17%, and 18%) for factor A and different energy levels (12.6 MJ/kg, 12.8 MJ/kg, and 13.0 MJ/kg) for factor B was used to design 12 groups of experimental diets for feeding. The experiment lasted for 28 d. We found that ME level had little effect on breeding pigeons, but the CP level and dietary energy/protein ratio significantly affected the reproductive and growth performance of the pigeons. The lowest total weight loss (*P* < 0.01), and the highest egg production (*P* < 0.01) were observed in group 11 (18% CP, 12.8 MJ/kg). It had no effect on egg quality. Both ME and CP levels significantly affected the growth performance, slaughter performance and meat quality of squabs, and there was a strong interaction between CP and ME. The fastest growth rate (*P* < 0.01) was observed in group 11 (18% CP, 12.8 MJ/kg). The best CP and ME combination for the eviscerated weight, pectoral muscle weight, organ weight, 45 min meat color (L^⁎^, a^⁎^, b^⁎^), pH, and muscle fiber characteristics were also group 11. Finally, the regression model revealed that the best dietary energy/protein ratio was 17.92 to 19.02 kcal/g for squabs and 16.72 kcal/g for the breeding pigeons. There was a strong interaction between energy and protein levels in breeding pigeons during the lactation period, and the best production performance was at 18% CP 12.8 MJ/kg. And this is recommended to be applied as the energy/protein ratio dietary requirement for breeding pigeons during lactation in the summer “2 + 4” pattern.

## INTRODUCTION

In recent years feed material prices around the world have continued to rise. Many breeding companies pay more attention to the effectiveness of feed inputs and outputs. Therefore, in the actual production of livestock and poultry breeding, providing accurate nutrition to animals can achieve the best production performance and breeding efficiency (Geng et al., 2022). The pigeon farming industry in China has developed rapidly, with annual production and sales of nearly 700 million squabs, which is the highest producer in the world (Kokoszynski et al., 2020). However, unlike other poultry, squabs rely heavily on the “crop milk” of their parents to meet their nutritional requirements for growth and development (Dong et al., 2012; Gillespie et al., 2012). Feeding nutritionally irrational diets may lead to malnutrition or overnutrition in broiler pigeons, resulting in poor reproductive performance of the parents and even reducing the survival rate of squabs (Xie et al., 2019). Therefore, meeting the nutritional needs of breeding pigeons is key to achieving optimal production and economic benefits. Many farms have recently adopted the “2 + 4” high-yield breeding pattern, in which a pair of breeding pigeons feeds 4 squabs simultaneously. This high-production model not only increases the breeder's feeding burden but also requires a higher nutritional supply for the parents during the lactation period.

Metabolizable energy (**ME**) and crude protein (**CP**) levels of diets are important factors affecting the growth, development, and reproductive performance of livestock and poultry (Liu et al., 2015; Zeng et al., 2015; Xia et al., 2019; Heijmans et al., 2021). ME and CP are not independent of each other. There is a clear link between them. When the level of CP and ME in the diet is maintained in an appropriate proportion, it is beneficial for the health status and production performance of poultry, etc. (Chen et al., 2021). Therefore, specifying the optimal energy-to-protein ratio nutritional level in diets is an important basis for other poultry science studies. Bu et al. (2015) found that optimal body weight and egg production performance could be achieved at a dietary CP level of 14.0% and an ME level of 12.30 MJ/kg for laying pigeons. It was also found that for optimal production performance of breeding pigeons, the optimal level of diet was 17% CP at 12.50 MJ/kg in the “2 + 2” breeding pattern and 18% CP at 12.50 MJ/kg in the “2 + 3” breeding pattern (Li et al., 2022). In previous studies on single forms of CP and ME levels, the optimal CP level required for breeding pigeons was about 16.16 to 18%, while the optimal ME level was about 11.85 to 12.81 MJ/kg (Ye et al., 2015; Gao et al., 2016a; Wang et al., 2018; Zhu et al., 2021). The huge variation in the study findings may be due to the differences in the experimental breeding patterns and seasonal variations, which make it difficult to be applied in production practice. In particular, the effect of the energy/protein ratio on the production of breeding pigeons during the lactation period is still unclear. Therefore, this study aimed to determine the optimal energy and protein ratio requirement of breeding pigeon diet during lactation under the "2 + 4" feeding pattern in summer by adopting two-way ANOVA experiment with different levels of CP and ME, so as to provide a reference for formulating the diet of breeding pigeon during lactation and large-scale breeding of meat pigeon.

## MATERIALS AND METHODS

All experimental procedures were carried out according to the Guidelines of Institutional Animal Care and approved by the Institutional Animal Ethics Committee of the Zhongkai University of Agricultural Engineering (Ethics Code: ZHKUMO-2021-075).

### Study Design

In this study, 576 pairs of Mimas white pigeons of 12 to 14 months old with similar weight and reproductive performance were selected to carry out a two-way experiment. The factors are 4 CP levels (15%, 16%, 17%, and 18%) and 3 ME levels (12.6 MJ/kg, 12.8 MJ/kg, and 13.0 MJ/kg), respectively. A total of 12 treatments were tested (Table 1). It reveals that the energy/protein ratios of the 1 to 12 treatment groups were 20.12 kcal/g, 20.34 kcal/g, 20.72 kcal/g, 18.86 kcal/g, 19.09 kcal/g, 19.42 kcal/g, 17.63 kcal/g, 18.00 kcal/g, 18.22 kcal/g, 16.72 kcal/g, 17.00 kcal/g, 17.26 kcal/g, respectively. During the experiment, the pigeons were randomly divided into 48 pairs with 8 replicates per treatment group. The experiment lasted for 28 d, including 21 d of lactation and 7 d of the resting period. Notably, the “2 + 4” breeding pattern was used. The experimental pigeons and squabs were provided by Guangdong Meizhou Golden Green Modern Agricultural Development Company. The breed is European Mimas white pigeon.

**Table 1:** Nutrient content of experimental diets for breeding pigeons during lactation.

Items	I	II	III	IV	V	VI	VII	VIII	IX	X	XI	XII
Corn	24.00	24.00	24.00	20.00	20.00	20.00	17.00	17.00	17.00	15.00	15.00	15.00
Sorghum	15.00	15.00	15.00	15.00	15.00	15.00	15.00	15.00	15.00	13.00	13.00	13.00
Wheat	15.00	15.00	15.00	16.50	16.50	16.50	16.00	16.00	16.00	13.00	13.00	13.00
Peas	41.00	39.00	37.00	39.00	37.00	35.50	39.00	37.00	35.00	43.50	41.50	39.50
Pal moil	0.00	1.00	2.00	0.80	1.80	2.80	1.50	2.50	3.50	2.30	3.30	4.30
Soybean meal	5.00	6.00	7.00	8.70	9.70	10.20	11.50	12.50	13.50	13.20	14.20	15.20
Total	100.00	100.00	100.00	100.00	100.00	100.00	100.00	100.00	100.00	100.00	100.00	100.00
Nutrient levels^1^												
CP (%)	14.98	15.05	15.02	15.97	16.04	16.02	17.08	17.00	17.05	18.00	17.98	18.02
EE (%)	2.25	3.23	4.22	2.72	3.71	4.69	3.21	4.17	5.14	3.67	4.56	5.63
Moisture (%)	12.26	12.14	12.02	12.22	12.11	11.99	12.20	12.07	11.95	12.16	12.05	11.93
Ash (%)	1.95	1.96	1.96	2.09	2.10	2.10	2.25	2.24	2.25	2.38	2.38	2.39
Ca (%)	0.08	0.08	0.08	0.09	0.09	0.09	0.10	0.10	0.10	0.11	0.11	0.11
TP (%)	0.34	0.34	0.34	0.35	0.35	0.35	0.36	0.36	0.36	0.37	0.37	0.36
ME (MJ/kg)	12.61	12.81	13.02	12.60	12.81	13.02	12.60	12.80	13.00	12.59	12.79	13.01
ME/CP (kcal/g)	20.12	20.34	20.72	18.86	19.09	19.42	17.63	18.00	18.22	16.72	17.00	17.26
SIDAA (%)												
Asp	1.42	1.43	1.43	1.53	1.55	1.55	1.67	1.66	1.67	1.78	1.78	1.79
Glu	2.84	2.84	2.81	2.99	2.97	2.96	3.14	3.12	3.13	3.27	3.26	3.26
Ser	0.72	0.72	0.72	0.76	0.77	0.77	0.82	0.81	0.82	0.86	0.86	0.86
His	0.38	0.39	0.39	0.41	0.41	0.41	0.44	0.43	0.44	0.46	0.46	0.46
Gly	0.55	0.55	0.55	0.56	0.56	0.56	0.57	0.57	0.56	0.58	0.57	0.57
Thr	0.54	0.55	0.55	0.58	0.58	0.58	0.62	0.62	0.62	0.66	0.66	0.66
Arg	1.12	1.13	1.13	1.19	1.20	1.20	1.27	1.27	1.27	1.34	1.34	1.34
Ala	0.71	0.71	0.71	0.74	0.75	0.74	0.79	0.78	0.78	0.82	0.82	0.82
Tyr	0.31	0.31	0.30	0.31	0.31	0.31	0.32	0.32	0.31	0.32	0.32	0.32
Val	0.57	0.57	0.57	0.62	0.62	0.62	0.67	0.67	0.67	0.72	0.72	0.72
Met	0.13	0.14	0.14	0.17	0.17	0.18	0.21	0.21	0.21	0.24	0.25	0.25
Phe	0.71	0.71	0.71	0.75	0.76	0.76	0.81	0.80	0.80	0.85	0.85	0.85
Ile	0.52	0.53	0.53	0.57	0.57	0.57	0.62	0.62	0.62	0.67	0.67	0.67
Leu	1.30	1.30	1.30	1.35	1.36	1.35	1.42	1.41	1.41	1.48	1.47	1.47
Lys	0.79	0.79	0.80	0.85	0.86	0.86	0.92	0.92	0.93	0.99	0.98	0.99

1 All nutrient levels were calculated values.

### Feeding and Routine Management

This study was carried out from July 2021 to September 2021. The temperature in the loft during the summer trials ranged from 23°C to 35°C with an average of 27°C to 28°C and the humidity ranged from 46 to 73% with an average of 75 to 76%. Each pair was kept in separate cage and provided with enough food and water. The single-cage system ensured the best care was provided for each pair. The pigeons were fed manually at regular intervals from 7:00 am, and every evening at 19:00 they were fasted. During experimental period, we checked the feed through 5 times a day to ensure that there was still material when the material was finally withdrawn. In this way, the daily feed intake can be accurately measured by weighing the added and remaining feed weight of each cage. The pigeons were fed on complete-formula granulated feeds. It was also supplemented with health sand prepared only from ordinary shells and gravel (in a ratio of 1:2) to help the breeding pigeons digest. The specific nutritional composition of the experimental feeds is shown in Table 1.

### Growth Performance of the Breeding Pigeons and Squabs During Lactation

The weights of the breeding pigeons before feeding and the 4 squabs nursed by each pair were weighed at 0 d, 7 d, 14 d, 21 d, and 28 d, respectively. The pigeons were counted separately by sex. The living condition of the squabs was observed and recorded daily promptly. The survival curve of the squabs during the whole period of the experiment was plotted based on the recorded data. Researchers recorded the daily additions and residuals to calculate the feed-to-gain ratio (**F/G**) of the squabs.

### Reproductive Performance of the Breeding Pigeons During Lactation

The egg weight, egg-laying interval, egg-laying rate, fertilization rate, and hatching rate in the first breeding cycle during lactation were recorded. The birth weights of the squabs were also recorded.

### Egg Quality During the Lactation Period

Thirty eggs in the second breeding cycle of lactation were randomly selected from each treatment group according to several similar studies (Zhang et al., 2021; Jin et al., 2022). Before measuring, all the eggs were stored at 4°C. The egg weight and yolk weight were determined using a universal electronic analytical balance (PWN124ZH/E). The egg shape index was calculated by measuring the longitudinal and transverse diameter with a vernier caliper (egg shape index = longitudinal diameter/transverse diameter). Eggshell strength was determined using a strength meter (EFR-01, ORKA Food Technology Ltd., Ramat Hasharon, Israel) with a blunt end for pigeon eggs. An automatic egg analyzer (EA-01, Robotmation Ltd., Tokyo, Japan) was used for determination at Haugh unit. The eggshells were washed with running water and dried naturally. Then the eggshells were weighed using a PWN124ZH/E 10,000-position electronic analytical balance. Finally, the shell membrane was removed to retain the calcified layer of eggshell. The thickness of the tip, middle and blunt end of eggshell was measured by a digital micrometer, and the average value of the 3 places was taken as the value of eggshell thickness.

### Slaughter Performance of Squabs

The squabs were fasted for 12 h at 21 d of age, and then 24 squabs were randomly sampled from each treatment group for slaughter. The slaughter weight, semieviscerated weight, eviscerated weight, pectoral muscle weight, abdominal fat weight, liver weight, heart weight, kidney weight, glandular stomach weight, gizzard weight, pancreas weight, spleen weight, thymus weight, and bursa weight of the slaughter squabs were determined according to the metric statistics method specified in *NY/T 823-2020 Poultry Production Performance Terms and Metric Statistical Methods* (Ministry of Agriculture of the People's Republic of China, 2020).

### Meat Physiochemical Quality of Slaughtered Squabs

The pectoral muscle samples were stored in sealed bags at 4°C after squab slaughtered for pH determination at 45 min and 24 h. The right pectoral muscle meat color (redness a^⁎^, yellowness b^⁎^, and lightness L^⁎^ values) and drip loss of the left pectoral muscle were determined according to the method of Petracci et al. (2013) at the stated intervals. Twelve squabs were randomly sampled from each treatment group of postslaughter pigeons. At the same time, about 1 cm^3^ muscle pieces were taken at the fixed position of the pectoral muscle and preserved in PFA fixative for backup. The fixed pectoral muscle samples were also sectioned (section thickness 10 μm) after gradient ethanol dehydration, xylene transparency, and paraffin embedding. These sections were rehydrated by gradient and stained using hematoxylin & eosin (**HE**). Then these stained sections were carefully observed and photographed under a 400× microscope. The muscle fiber area and muscle fiber diameter of individual muscle fibers in each photograph were measured using Image J software. The number of muscle fibers in 100*100 μm^2^ was marked according to the method adopted by Chen et al. (2020) using the counting tool in Image J software. And finally converted to the density of muscle fibers in a 1 mm^2^ photograph. The final data of muscle fiber traits were obtained for 12 groups for subsequent analysis.

### Statistical Analysis

The data were analyzed using Excel and SPSS software, version 26.0. Differences between groups were compared using the Duncan's method, while the interaction between ME and CP was analyzed by 2-way ANOVA based on the general linear model (**GLM**). The significance test was performed using the LSD method, and *P* < 0.05 was considered statistically significant. The corresponding data were analyzed by linear and quadratic regression analysis of energy/protein ratio requirements.

## RESULTS

### Dietary Energy/Protein Ratio Affected the Growth Performance of Breeding Pigeons

High CP levels significantly reduced the weight loss of the breeding pigeons during lactation. Meanwhile, the weight loss of the breeding pigeons was lowest at 18% CP, which is significantly lower than in the other CP groups (*P* < 0.01). The ME level had little effect on the weight of the breeding pigeons. There was a strong interaction between CP and ME, which jointly affected the weight of the breeding pigeons during the lactation period. Group 12 (18% CP, 13.0 MJ/kg) had the lowest weight loss during lactation and the fastest weight recovery after the lactation period when the dietary energy/protein ratio was 17.26 kcal/g (Table 2).

**Table 2:** Effect of dietary energy/protein ratios on the growth performance of breeding pigeons during lactation in summer.

Treatments (*n* = 8)	CP/%	ME/MJ/kg	Male pigeon weight loss/g	Female pigeon weight loss/g	Total weight loss/g
I	15	12.6	91.262CDE	68.700^CD^	159.962DEF
II	15	12.8	102.725ABCD	74.300^CD^	177.025CDEF
III	15	13.0	116.850^AB^	87.025ABC	203.875ABC
IV	16	12.6	108.575ABC	76.100^CD^	184.675BCD
V	16	12.8	107.812ABCD	69.788^CD^	177.538CDEF
VI	16	13.0	123.525^A^	102.375^A^	225.900^A^
VII	17	12.6	119.275^AB^	97.900^AB^	217.288^AB^
VIII	17	12.8	113.562^AB^	69.550^CD^	183.288CDE
IX	17	13.0	88.825CDE	61.213^D^	150.050^EF^
X	18	12.6	76.550^E^	79.075BCD	156.775DEF
XI	18	12.8	98.375BCDE	61.763^D^	155.613DEF
XII	18	13.0	86.230^DE^	64.210^D^	149.710^F^
SEM			2.673	2.364	3.994
Main effect					
CP (*n* = 24)	15%		103.612^A^	76.675	180.287^A^
	16%		113.304^A^	82.754	196.037^A^
	17%		107.221^A^	76.221	183.542^A^
	18%		87.495^B^	67.663	153.852^B^
ME (*n* = 32)		12.6 MJ/kg	98.916	80.444	179.675
		12.8 MJ/kg	105.619	68.850	173.366
		13.0 MJ/kg	104.190	78.191	182.249
*P* values		CP	<0.001	0.126	<0.001
		ME	0.457	0.059	0.547
		C*M	<0.001	<0.001	<0.001

A-F Different superscript capital letters indicate extremely significant differences (*P* < 0.01), *n* = 8/group; C*M CP × ME; Total weight loss = (Male + Female) pigeon weight loss/g.

### Dietary Energy/Protein Ratio Affected the Reproductive Performance of Breeding Pigeons

The interaction of dietary ME levels and CP levels had a significant effect on breeding pigeons' reproductive performance during the lactation period. The CP level positively correlated with the laying rate during the lactation period. The shortest average laying interval and the highest laying rate were observed at a CP rate of 18% (*P* < 0.01). The best reproductive performance was observed in groups 9 (17% CP, 13.0 MJ/kg) and 10 (18% CP, 12.6 MJ/kg) (*P* < 0.01), followed by group 11 (18% CP, 12.8 MJ/kg) (Table 3). In other words, the breeding efficiency can be improved when the ratio of energy/protein of 18.22 kcal/g and 16.72 kcal/g, followed by 17.00 kcal/g.

**Table 3:** Effect of dietary energy/protein ratios on the reproductive performance of breeding pigeons during lactation in summer.

Treatments (*n* = 8)	CP/%	ME/MJ/kg	Average laying interval/d	Average egg weight/g	Fertility rate/%	Hatchability/%	Average birth weight/g	45-day laying rate/%	50-day laying rate/%	55-day laying rate/%
I	15	12.6	50.150ABC	22.993	91.706	88.060	14.680	12.500	37.499^CD^	68.749
II	15	12.8	50.670ABC	23.596	89.029	86.078	15.152	12.501	39.581^CD^	77.082
III	15	13.0	50.596ABC	24.759	86.285	78.959	14.642	12.500	41.668^CD^	79.166
IV	16	12.6	51.883^AB^	24.413	86.001	86.001	15.765	8.335	27.084^D^	72.916
V	16	12.8	50.018ABC	24.057	96.825	96.825	14.902	12.500	54.169^BC^	77.081
VI	16	13.0	52.271^AB^	22.722	94.463	84.444	14.851	10.416	33.334^CD^	70.834
VII	17	12.6	52.775^A^	22.662	96.875	94.236	14.703	6.250	22.918^D^	64.582
VIII	17	12.8	50.621ABC	23.268	97.500	88.750	14.867	17.085	40.832^CD^	72.500
IX	17	13.0	48.020^C^	23.054	96.086	89.960	14.877	20.834	81.249^A^	95.833
X	18	12.6	47.696^C^	22.677	92.103	84.504	14.742	15.416	77.915^A^	88.749
XI	18	12.8	49.121^BC^	23.383	92.311	90.923	14.879	23.334	73.749^AB^	78.332
XII	18	13.0	49.175^BC^	23.848	96.875	92.266	15.560	23.750	55.000^BC^	87.500
SEM			0.309	0.210	1.029	1.247	0.076	1.610	2.823	2.096
Main effect										
CP (*n* = 24)	15%		50.472^A^	23.782	89.007	84.365	14.825	12.500	39.583^B^	74.999
	16%		51.391^A^	23.731	92.430	89.090	15.173	10.417	38.195^B^	73.610
	17%		50.472^A^	22.995	96.820	90.982	14.816	14.723	48.333^B^	77.638
	18%		48.664^B^	23.303	93.763	89.231	15.060	20.833	68.888^A^	84.860
ME (*n* = 32)		12.6 MJ/kg	50.626	23.186	91.671	88.200	14.972	10.625	41.354	73.749
		12.8 MJ/kg	50.108	23.576	93.916	90.644	14.950	16.355	52.083	76.249
		13.0 MJ/kg	50.016	23.596	93.428	86.407	14.982	16.875	52.813	83.333
*P* values		CP	0.010	0.492	0.058	0.268	0.985	0.126	<0.001	0.211
		ME	0.644	0.679	0.632	0.371	0.236	0.218	0.061	0.139
		C*M	0.008	0.527	0.178	0.232	0.091	0.432	<0.001	0.094

A-D Different superscript capital letters indicate extremely significant differences (*P* < 0.01), *n* = 8/group; C*M CP × ME.

### Dietary Energy/Protein Ratio Affected the Egg Quality of Breeding Pigeons

Quality test results of the second breeding cycle of eggs laid by breeding pigeons after the start of lactation are shown in Table 4, which showed that CP and ME levels had little effect on the egg quality of breeding pigeons in summer (*P* > 0.05), and there was no significant interaction between CP and ME on their effect on egg quality (*P* > 0.05).

**Table 4:** Effect of dietary energy/protein ratios on egg production quality of breeding pigeons during lactation in summer.

Treatments (*n* = 8)	CP/%	ME/MJ/kg	Egg weight/g	Relative yolk weight/g	Relative shell weight/g	Egg shape index	Shell strength/Pa	Shell thickness/mm	Haugh unit
I	15	12.6	23.024	4.510	1.640	1.378	11.014	0.223	76.306
II	15	12.8	23.396	4.468	1.615	1.399	10.126	0.224	75.982
III	15	13.0	22.848	4.428	1.579	1.383	10.606	0.220	78.400
IV	16	12.6	24.432	4.482	1.679	1.468	10.786	0.222	77.433
V	16	12.8	23.404	4.457	1.596	1.392	10.196	0.220	76.194
VI	16	13.0	23.336	4.356	1.638	1.395	10.899	0.227	76.910
VII	17	12.6	22.520	4.348	1.585	1.486	11.210	0.225	75.418
VIII	17	12.8	23.652	4.486	1.631	1.375	10.168	0.219	77.852
IX	17	13.0	23.540	4.612	1.622	1.379	10.978	0.216	76.535
X	18	12.6	22.248	4.424	1.546	1.391	11.042	0.213	76.763
XI	18	12.8	23.404	4.558	1.619	1.388	11.419	0.219	77.040
XII	18	13.0	23.560	4.617	1.624	1.390	11.397	0.220	77.112
SEM			0.130	0.031	0.009	0.003	0.115	0.001	0.529
Main effect									
CP (*n* = 24)	15%		23.089	4.469	1.611	1.387	10.582	0.222	76.896
	16%		23.724	4.431	1.638	1.418	10.627	0.223	76.846
	17%		23.237	4.482	1.613	1.447	10.785	0.220	76.601
	18%		23.071	4.533	1.596	1.390	11.286	0.217	76.971
ME (*n* = 32)		12.6 MJ/kg	23.056	4.441	1.613	1.431	11.013	0.221	76.480
		12.8 MJ/kg	23.464	4.492	1.615	1.388	10.477	0.220	76.767
		13.0 MJ/kg	23.321	4.503	1.616	1.437	10.970	0.221	77.239
*P* values		CP	0.241	0.281	0.477	0.797	0.118	0.302	0.968
		ME	0.422	0.278	0.990	0.147	0.106	0.944	0.539
		C*M	0.090	0.311	0.310	0.689	0.200	0.433	0.699

The absence of superscript letters in the same row of data indicates that the difference is not significant (*P* > 0.05), *n* = 8/group; C*M CP × ME.

### Dietary Energy/Protein Ratio Affected the Growth Performance of Squabs

**Figure 1:**
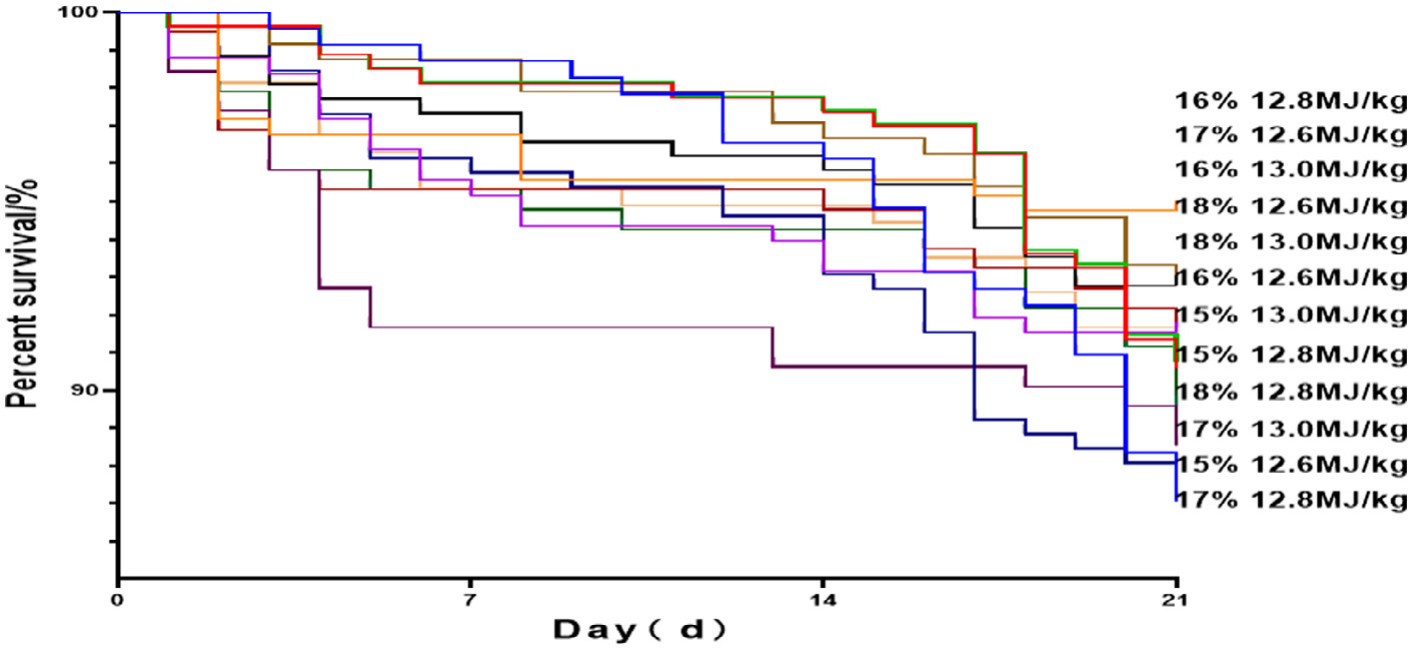
Full-term survival curve of pigeon squabs in different energy/protein ratios diet groups. The treatment groups on the right were ranked according to the squab survival rate at the end of the experiment.

Figure 1 shows the survival rates of the squabs during the whole experimental period. The survival rate of squabs in all groups during the lactation period decreased with an increase in age, but CP and ME levels had little effect on this phenomenon (*P* > 0.05). There was no significant interaction between CP and ME on their effect on the survival rates of the squabs (*P* > 0.05).

In the early and middle growth stages (1–14 d), squab weight and average litter weight increased and then decreased with a further increase in CP levels. The fastest growth rate was observed at a CP content of 16% (*P* < 0.01). In the later growth stage (14–21 d), 18% CP was the optimal proportion for the nutritional needs of the squabs (*P* < 0.01). The ME level only affected the early growth stages of the squab (1–7 d), and the highest weight gain was observed at 12.8 MJ/kg (*P* < 0.05). The interaction between CP and ME had a significant effect on squab growth, and the highest squab weight gain (*P* < 0.01) during the lactation period was observed in groups 5 (17% CP, 13.0 MJ/kg) and 11 (18% CP, 12.8 MJ/kg). The average feed intake of breeding pigeons increased significantly (*P* < 0.01) with CP levels and a decrease in ME levels. Group 1 (15% CP, 12.6 MJ/kg) had the highest feed intake during the whole study period (*P* < 0.01). Combining with the litter weight gain of the squabs, we found that group 5 (16% CP, 12.8 MJ/kg) with energy/protein ratio of 19.09 kcal/g had the lowest full-term F/G and the highest production benefit (*P* < 0.01) (Table 5).

**Table 5:** Effect of dietary energy/protein ratios on the growth performance of squabs in summer.

Treatments (*n* = 8)	CP/%	ME/MJ/kg	0-day SW/g	7-day SW/g	14-day SW/g	21-day SW/g	0-day LW/g	7-day LW/g	14-day LW/g	21-day LW/g	Total feed intake/g	First week F/G	Second week F/G	Third week F/G	Full-term F/G
I	15	12.6	16.037	105.526^BC^	243.316ABC	351.234^AB^	64.149	422.666^CD^	964.282^AB^	1400.532^A^	3727.765^A^	1.487^DE^	2.503^CD^	3.957DEF	2.740BCD
II	15	12.8	15.790	108.341^B^	231.666CDE	277.831^F^	63.159	435.918^BC^	937.015BCD	1135.606^E^	3370.025^CD^	1.633BCD	2.837ABC	6.620^A^	3.147^A^
III	15	13.0	16.135	98.989^C^	222.769^E^	287.684^EF^	64.534	396.794^D^	889.843^D^	1175.340^DE^	3082.219^E^	1.523CDE	2.565^CD^	5.144^BC^	2.784^BC^
IV	16	12.6	15.933	103.310^BC^	248.335^AB^	326.999^CD^	63.726	408.135^CD^	996.613^AB^	1308.000^BC^	3481.571ABC	1.810^A^	2.328^D^	5.308^BC^	2.814^BC^
V	16	12.8	16.124	116.981^A^	251.740^A^	352.593^A^	64.498	474.913^A^	1007.270^A^	1413.779^A^	3115.514^E^	1.369^E^	2.432^D^	3.445^EF^	2.311^E^
VI	16	13.0	16.237	114.629^AB^	249.633^A^	322.917^D^	64.948	462.066^AB^	996.590^AB^	1297.692^C^	3162.103^DE^	1.569^CD^	2.342^D^	4.596^CD^	2.565^D^
VII	17	12.6	16.064	100.134^C^	232.767BCDE	289.662^EF^	64.259	405.000^CD^	938.550BCD	1170.774^DE^	3204.474^DE^	1.736^AB^	2.443^D^	5.881^AB^	2.901^B^
VIII	17	12.8	16.126	103.616^BC^	224.261^DE^	302.463^E^	64.500	418.435^CD^	894.975^CD^	1217.481^D^	3089.408^E^	1.668ABC	2.691BCD	4.270CDE	2.688BCD
IX	17	13.0	16.173	99.319^C^	238.958ABCD	331.998BCD	64.693	395.244^D^	958.450ABC	1323.750^BC^	3502.365ABC	1.483^DE^	2.484^CD^	4.528^CD^	2.783^BC^
X	18	12.6	16.036	100.503^BC^	221.541^E^	323.387^D^	64.136	403.925^CD^	895.406^CD^	1316.762^BC^	3628.470^AB^	1.597BCD	3.064^A^	3.818^DE^	2.900^B^
XI	18	12.8	16.059	104.411^BC^	224.679^DE^	352.049^A^	64.231	417.543^CD^	894.933^CD^	1422.587^A^	3584.260ABC	1.664ABC	3.047^AB^	3.173^F^	2.640^CD^
XII	18	13.0	16.228	103.389^BC^	218.069^E^	344.090ABC	64.900	413.554^CD^	872.271^D^	1372.329^AB^	3399.979BCD	1.552^CD^	3.044^AB^	3.039^F^	2.605^CD^
SEM			0.171	3.967	7.968	12.397	0.043	0.959	1.952	3.255	32.923	0.019	0.045	0.150	0.029
Main effect															
CP (*n* = 24)	15%		15.988	104.285^B^	232.584^B^	305.584^B^	63.947	418.459^B^	930.379^B^	1237.159^B^	3393.336^AB^	1.548	2.635^B^	5.240^A^	2.890^A^
	16%		16.098	111.640^A^	249.903^A^	334.170^A^	64.390	448.4371^A^	1000.157^A^	1339.824^A^	3253.063^B^	1.583	2.367^C^	4.450^B^	2.563^C^
	17%		16.121	101.023^B^	231.995^B^	308.041^B^	64.484	406.226^B^	930.658^B^	1237.335^B^	3265.415^B^	1.629	2.539^BC^	4.893^AB^	2.791^AB^
	18%		16.108	102.767^B^	221.430^C^	339.842^A^	64.423	411.674^B^	887.537^C^	1370.560^A^	3537.570^A^	1.604	3.052^A^	3.343^B^	2.715^B^
ME (*n* = 32)		12.6 MJ/kg	16.018	102.368^b^	236.490	322.821	64.068	409.931^B^	948.713	1299.0167	3510.570^A^	1.658^A^	2.584	4.741	2.839^A^
		12.8 MJ/kg	16.025	108.337^a^	233.087	321.234	64.097	436.702^A^	933.548	1297.363	3289.802^B^	1.583^AB^	2.752	5.377	2.696^B^
		13.0 MJ/kg	16.193	104.081^b^	232.357	321.672	64.768	416.914^B^	929.288	1292.278	3286.666^B^	1.532^B^	2.609	4.327	2.684^B^
*P* values		CP	0.689	<0.001	<0.001	<0.001	0.684	<0.001	<0.001	<0.001	<0.001	0.346	<0.001	<0.001	<0.001
		ME	0.183	0.012	0.553	0.948	0.183	0.004	0.478	0.932	0.001	0.009	0.148	0.258	0.009
		C*M	0.747	<0.001	<0.001	<0.001	0.745	<0.001	<0.001	<0.001	<0.001	<0.001	<0.001	0.001	<0.001

a-b Different superscript lowercase letters in the data of the same row indicate significant differences (*P* < 0.05), and ^A-F^different superscript capital letters indicate extremely significant differences (*P* < 0.01), *n* = 8/group; C*M CP × ME; SW, squab weight; LW, average litter weight; F/G, feed-to-gain ratio.

### Dietary Energy/Protein Ratio Affected the Slaughter Performance of Squabs

Both ME and CP levels significantly affected the slaughter performance of squabs, and the semieviscerated weight, eviscerated weight, and heart weight of the squabs decreased with an increase in ME level (*P* < 0.05). Compared with other CP levels, the slaughter weight, semieviscerated weight, eviscerated weight, abdominal fat, and gizzard weights were significantly high at 18% CP level (*P* < 0.05). The interaction between ME and CP significantly affected the slaughter weight, semieviscerated weight, eviscerated weight, pectoral muscle weight, abdominal fat, gizzard, and kidney weights. Group 11 (18% CP, 12.8 MJ/kg) with an energy/protein ratio of 17.00 kcal/g had the best body fat and organ development (*P* < 0.05) (Table 6).

**Table 6:** Effect of dietary energy/protein ratios on the slaughter performance of squabs in summer.

Treatments (*n* = 24)	CP/%	ME/MJ/kg	Live body weight/g	Slaughter weight/g	Semieviscerated weight/g	Eviscerated weight/g	Pectoral muscle weight/g	Abdominal fat/g	Liver/g	Spleen/g	Thymus/g	Pancreas/g	Glandular stomach/g	Gizzard/g	Kidney/g	Bursa/g	Heart/g
I	15	12.6	369.688^AB^	289.858^ab^	168.863^AB^	103.046^AB^	26.507^ab^	1.538^b^	12.150	0.383	0.932	2.276	1.253	6.504ABC	2.566^c^	0.493^AB^	3.315^A^
II	15	12.8	301.225^E^	246.220^c^	140.515^D^	82.906^D^	19.857^c^	1.688^ab^	10.498	0.297	0.827	2.029	1.228	6.108^CD^	2.871abc	0.285^C^	2.718^BC^
III	15	13.0	301.068^E^	242.827^c^	144.043^CD^	84.931^CD^	23.211abc	1.680^ab^	10.693	0.311	1.178	2.104	1.196	5.694^CD^	2.852abc	0.373^BC^	2.619^C^
IV	16	12.6	309.458^DE^	253.233^bc^	152.304ABCD	93.241ABCD	24.062abc	2.573^a^	11.289	0.293	1.167	2.185	1.103	5.683^CD^	2.742abc	0.323^C^	2.767^BC^
V	16	12.8	334.000BCD	272.171^ab^	155.913ABCD	98.410ABC	21.904abc	1.718^ab^	11.683	0.404	0.965	2.186	1.221	5.843^CD^	2.549^c^	0.533^A^	2.982^AB^
VI	16	13.0	309.300^DE^	261.105^bc^	143.213^CD^	84.439^CD^	19.077^c^	1.907^ab^	10.001	0.369	0.747	2.094	1.111	5.424^D^	2.571^bc^	0.324^C^	2.702^BC^
VII	17	12.6	343.591ABCD	278.518^ab^	160.291ABC	100.977^AB^	25.525^ab^	1.936^ab^	11.144	0.321	1.435	2.522	1.270	6.186^BC^	3.151^a^	0.376^BC^	2.859^BC^
VIII	17	12.8	329.100BCDE	261.725^bc^	147.820BCD	89.794BCD	23.276abc	2.102^ab^	10.006	0.289	1.247	2.143	1.288	6.089^CD^	3.033abc	0.380^BC^	2.623^C^
IX	17	13.0	325.350CDE	274.135abc	151.530ABCD	91.752ABCD	21.735^bc^	1.862^ab^	10.717	0.306	0.488	2.116	1.238	6.005^CD^	2.594^bc^	0.331^C^	2.778^BC^
X	18	12.6	336.238BCD	279.357^ab^	165.656^A^	104.814^A^	23.568abc	2.341^ab^	11.473	0.332	0.824	2.266	1.178	6.207^BC^	2.609abc	0.345^C^	2.975^BC^
XI	18	12.8	360.700ABC	287.400^ab^	169.811^AB^	103.807^A^	27.849^a^	2.556^a^	11.416	0.331	1.258	2.459	1.306	7.078^A^	3.106^ab^	0.54^BC^	2.796^BC^
XII	18	13.0	383.700^A^	295.775^a^	161.720ABC	99.531^AB^	25.871^ab^	2.263^ab^	10.789	0.297	1.356	2.278	1.361	6.848^AB^	2.994abc	0.418^BC^	2.912^BC^
SEM			3.823	3.020	1.870	1.469	0.535	0.087	0.149	0.009	0.045	0.037	0.021	0.072	0.050	0.015	0.035
Main effect																	
CP (*n* = 72)	15%		323.008^B^	259.020^B^	150.397^b^	90.294^b^	23.043	1.651^b^	10.993	0.328	0.984	2.133	1.248	6.150^B^	2.739	0.390	2.904
	16%		323.933^B^	267.085^B^	150.154^b^	92.029^b^	22.331	2.239^a^	11.159	0.363	1.026	2.164	1.146	5.756^C^	2.650	0.410	2.836
	17%		334.050^B^	272.552^B^	153.797^b^	94.174^b^	23.653	1.969^ab^	10.669	0.307	1.072	2.260	1.274	6.118^BC^	2.927	0.365	2.763
	18%		361.733^A^	288.753^A^	165.397^a^	102.717^a^	25.976	2.386^a^	11.263	0.322	1.155	2.338	1.287	6.724^A^	2.903	0.372	2.900
ME (*n* = 96)		12.6 MJ/kg	343.325	278.309	163.459^A^	100.519^A^	25.296	2.184	11.638^A^	0.339	1.137	2.306	1.229	6.224	2.786	0.393	2.990^a^
		12.8 MJ/kg	333.919	268.786	153.630^B^	93.729^ab^	23.520	2.058	10.881^B^	0.331	1.098	2.204	1.252	6.300	2.877	0.398	2.801^b^
		13.0 MJ/kg	329.800	268.463	149.960^B^	90.163^b^	22.436	1.942	10.544^B^	0.319	0.942	2.161	1.236	6.037	2.751	0.362	2.760^b^
*P* values		CP	<0.001	0.003	0.019	0.011	0.079	0.014	0.504	0.168	0.517	0.179	0.065	<0.001	0.141	0.695	0.407
		ME	0.280	0.290	0.008	0.011	0.080	0.509	0.009	0.674	0.134	0.248	0.895	0.266	0.558	0.542	0.013
		C*M	<0.001	0.020	0.004	0.003	0.021	0.050	0.073	0.163	0.661	0.217	0.406	<0.001	0.027	0.003	0.001

a-c Different superscript lowercase letters in the data of the same row indicate significant differences (*P* < 0.05), and ^A-E^different superscript capital letters indicate extremely significant differences (*P* < 0.01), *n* = 24/group; C*M CP × ME.

### Dietary Energy/Protein Ratio Affected the Meat Physiochemical Quality of Slaughtered Squabs

Both ME and CP levels also had a significant effect on the physicochemical traits of the pectoral muscle of the squabs. Furthermore, there was a strong interaction between ME and CP levels regarding the meat quality of the pectoral muscle of the squabs. Overall, 18% CP affected the meat color the most (lightness L^⁎^, redness a^⁎^, yellowness b^⁎^) at 45 min and pH after slaughter (*P* < 0.01). The lowest pH after 24 h was observed in the 12.8 MJ/kg group (*P* < 0.05) (Table 7).

**Table 7:** Effect of dietary energy/protein ratios on the meat quality of squabs in summer.

Treatments (*n* = 24)	CP/%	ME/MJ/kg	45-min L^⁎^	45-min a^⁎^	45-min b^⁎^	pH 45 min	pH 24 h	Drip loss/%
I	15	12.6	41.614^CD^	9.995ABC	8.881ABCD	6.223^A^	5.822ABCD	1.989^ab^
II	15	12.8	42.674^CD^	10.068^AB^	8.961ABC	6.156^A^	5.787^CD^	1.660^ab^
III	15	13.0	47.775^A^	9.157BCD	9.256^AB^	6.189^A^	5.932^AB^	2.214^a^
IV	16	12.6	42.919^CD^	9.170BCD	8.417ABC	6.118^AB^	5.946^A^	1.736^ab^
V	16	12.8	41.535^CD^	11.276^A^	9.814^A^	6.287^A^	5.878ABCD	2.014^a^
VI	16	13.0	42.557^CD^	9.553ABCD	8.080ABCD	6.172^A^	5.862ABC	1.255^b^
VII	17	12.6	44.351^BC^	8.446BCD	7.014^D^	5.980^BC^	5.800BCD	2.077^a^
VIII	17	12.8	46.386^AB^	8.148^CD^	8.698ABC	6.096ABC	5.938^A^	1.603^ab^
IX	17	13.0	42.933^CD^	8.663BCD	7.274^CD^	6.237^A^	5.821^A^	1.250^b^
X	18	12.6	44.100BCD	8.110^D^	7.518^CD^	6.118^AB^	5.848ABCD	1.525^ab^
XI	18	12.8	42.458^D^	8.617BCD	7.010BCD	5.955^C^	5.721^D^	1.548^ab^
XII	18	13.0	43.874BCD	8.429BCD	6.983^CD^	6.114^AB^	5.850ABCD	1.586^ab^
SEM			0.284	0.131	0.139	0.015	0.013	0.061
Main effect								
CP (*n* = 72)	15%		44.129^a^	9.728^A^	9.002^A^	6.201^A^	5.851^ab^	1.954
	16%		42.462^b^	9.806^A^	8.677^A^	6.183^A^	5.883^a^	1.669
	17%		44.620^a^	8.396^B^	7.575^B^	6.108^B^	5.894^a^	1.643
	18%		43.419^ab^	8.351^B^	7.070^B^	6.064^B^	5.801^b^	1.666
ME (*n* = 96)		12.6 MJ/kg	43.398	8.864	7.809^b^	6.122	5.854^ab^	1.832
		12.8 MJ/kg	43.257	9.410	8.528^a^	6.114	5.820^b^	1.706
		13.0 MJ/kg	44.318	8.936	7.905^b^	6.181	5.898^a^	1.661
*P* values		CP	0.026	<0.001	<0.001	0.003	0.031	0.155
		ME	0.209	0.145	0.048	0.121	0.034	0.280
		C*M	<0.001	<0.001	<0.001	<0.001	0.001	0.039

a-b Different superscript lowercase letters in the data of the same row indicate significant differences (*P* < 0.05), and ^A-D^different superscript capital letters indicate extremely significant differences (*P* < 0.01), *n* = 24/group; C*M CP × ME; L^⁎^ stands for brightness; a^⁎^ stands for redness; b^⁎^ stands for yellowness.

**Figure 2:**
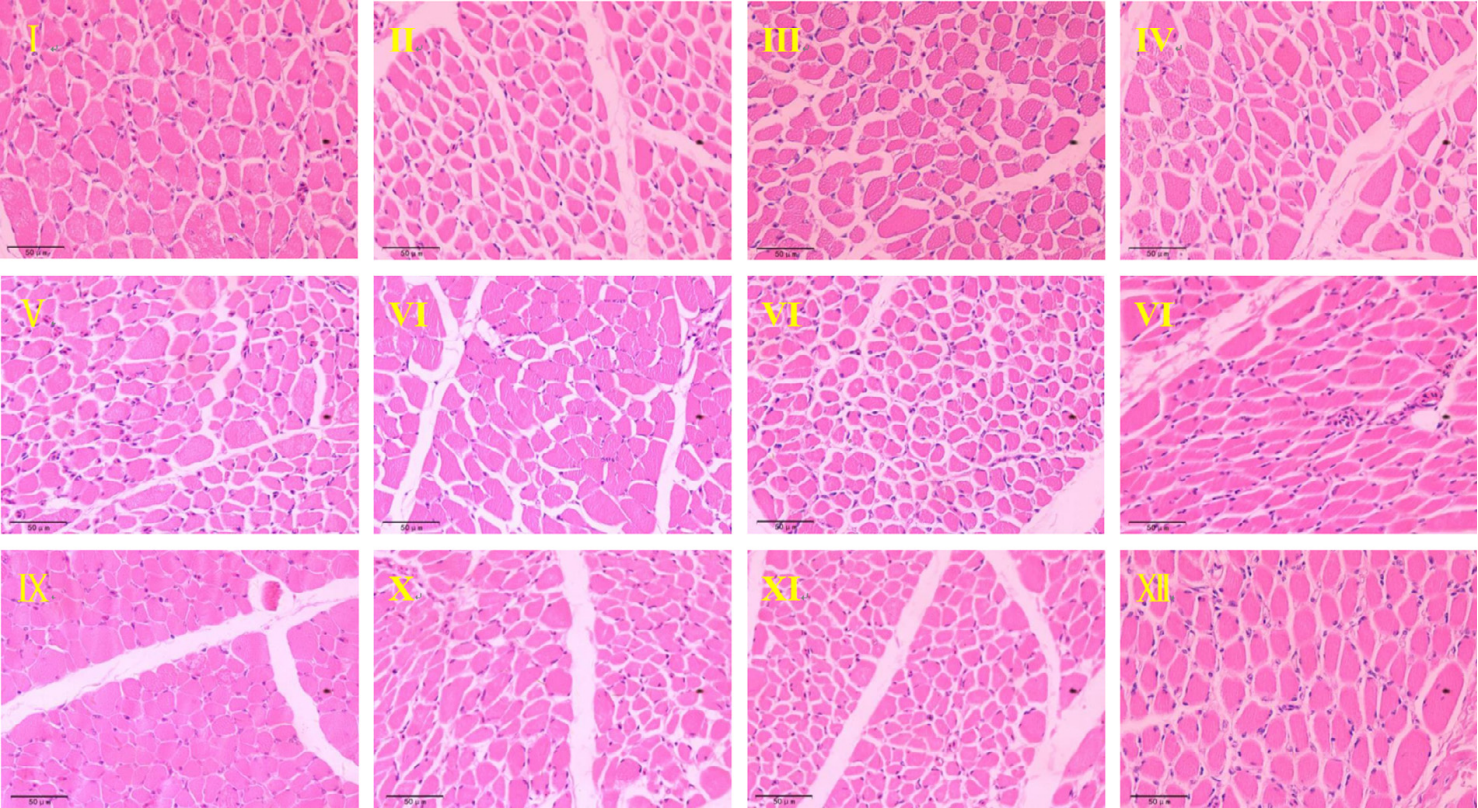
Effect of dietary energy/protein ratio on the muscle fiber development of slaughter squabs (400×). The Roman numerals I to XII at the top left of the image represent different processing groups.

Further investigation revealed that the ME level had a significant effect on the tissue biological properties of muscle fiber in pigeons, especially in the 12.8 MJ/kg group, which had the smallest cross-sectional area and diameter of muscle fiber (*P* < 0.01) and the highest muscle fiber density (*P* < 0.01) (Table 8). As shown in Figure 2, there was also a significant interaction between ME and CP levels. Compared to the other test groups, test group 11 (18% CP, 12.8 MJ/kg) significantly increased the muscle fiber density and decreased the cross-sectional area of pectoral muscle fibers (*P* < 0.01). Therefore, 18% CP and 12.8 MJ/kg was the optimal combination to improve the quality of pigeon breast with a corresponding energy/protein ratio of 17.00 kcal/g.

**Table 8:** Effect of dietary energy/protein ratios on the muscle fiber characteristics of squabs in summer.

Treatments (*n* = 12)	CP/%	ME/MJ/kg	Muscle fiber area/μm^2^	Muscle fiber diameter/μm	Muscle fiber density/n*mm^−2^
I	15	12.6	336.134^A^	20.455^A^	2108.333^D^
II	15	12.8	242.099^DE^	17.167^DE^	3358.333^A^
III	15	13.0	257.148CDE	17.644CDE	2983.333^AB^
IV	16	12.6	277.801BCD	18.391BCD	2358.333BCD
V	16	12.8	238.056^DE^	17.135^DE^	2808.333ABC
VI	16	13.0	273.971BCD	18.390BCD	2891.667ABC
VII	17	12.6	247.163CDE	17.549CDE	3100.000^A^
VIII	17	12.8	258.318CDE	17.775CDE	3225.000^A^
IX	17	13.0	259.244CDE	18.023BCD	2850.000ABC
X	18	12.6	281.403ABCD	18.550ABCD	2300.000^CD^
XI	18	12.8	208.752^E^	15.920^E^	3433.333^A^
XII	18	13.0	302.236ABC	19.294ABC	2000.000^D^
SEM			6.179	0.214	76.632
Main effect					
CP (*n* = 36)	15%		278.461	18.422	2816.667
	16%		263.276	17.972	2686.111
	17%		254.908	17.782	3058.333
	18%		264.130	17.921	2577.778
ME (*n* = 48)		12.6 MJ/kg	285.625^A^	18.736^A^	2466.667^B^
		12.8 MJ/kg	236.806^B^	16.999^B^	3206.250^A^
		13.0 MJ/kg	273.149^A^	18.338^A^	2681.250^B^
*P* values		CP	0.551	0.701	0.084
		ME	0.002	0.002	<0.001
		C*M	0.004	0.004	<0.001

A-E Different superscript capital letters indicate extremely significant differences (*P* < 0.01), *n* = 12/group;C*M CP × ME.

### Regression Models to Estimate the Dietary Energy/Protein Ratio Requirements of Breeding Pigeons

The 1 to 21-day ADG, slaughter rate, drip loss, the 50-day laying rate, total weight loss of the breeding pigeons, average laying interval, and full-term F/G all showed significant quadratic curve changes with the change of energy/protein ratio level in the dietary. As shown in Table 9, the optimal energy/protein ratio for lactating breeding pigeons in summer varies according to different indices. For the slaughter rate and F/G of squabs, the optimal dietary energy/protein ratio was 17.919 to 19.020 kcal/g; for the laying period and weight recovery after lactation, the optimal dietary energy/protein ratio for breeding pigeons was 16.720 kcal/g.

**Table 9:** Regression analysis of dietary energy/protein ratio requirements for lactating pigeons.

Items	Regression model	Coefficient (*R*^2^)	*P* value	Dietary energy/protein ratio additive amount/(kcal/g)
Pigeon squabs 1–21-day ADG /g	*y* = 21.235 − 0.319*x*	0.068	0.010	17.919 (max)
	*y* = −0.203*x*^2^ + 7.275*x* − 49.465	0.081	0.007	
Slaughter rate/%	*y* = 75.591 + 0.294*x*	0.004	0.308	19.020 (max)
	*y* = −0.510*x*^2^ + 19.400*x* − 102.379	0.012	0.094	
Drip loss/%	*y* = 0.066 + 0.001*x*	0.008	0.749	19.188 (min)
	*y* = 0.008*x*^2^ − 0.307*x* + 2.945	0.009	0.129	
The 50-day laying rate/%	*y* = 186.834 − 7.418x	0.122	<0.001	19.665 (min)
	*y* = 3.877*x*^2^ − 152.482*x* + 1537.245	0.161	<0.001	
Total heavy loss of breeding pigeons/g	*y* = 40.410 + 7.417*x*	0.057	0.019	19.731 (max)
	*y* = −3.622*x*^2^ + 142.930*x* − 1221.90	0.054	0.029	
Average laying interval/d	*y* = 41.461 + 0.473*x*	0.040	0.049	19.289 (max)
	*y* = −0.407*x*^2^ + 15.701*x* − 100.290	0.056	0.025	
Full-term F/G	*y* = 2.403 + 0.018*x*	0.007	0.424	18.404 (min)
	*y* = 0.057*x*^2^ − 2.098*x* + 22.102	0.066	0.016	

## DISCUSSION

Appropriate levels of ME and CP ensure the nutritional quality of the diet and have important implications for promoting poultry growth and development, immune function, and reproduction of offspring (Ye et al., 2015). Lesuisse et al. (2017, 2018) obtained a higher energy/protein ratio by reducing the CP level in the diet, which resulted in a higher body fat ratio during the actual production of breeding pigeons. And such a higher body fat amount obtained by consuming less CP may be detrimental to the breeder's productive performance (Heijmans et al., 2021). If the energy/protein ratio of the dietary is not balanced, it will directly affect the growth and reproductive performance of breeding pigeons. Herein, we first evaluated the effects of different energy/protein levels on the growth and reproductive performance, “crop milk” quality, and egg quality of breeding pigeons. We found that as the dietary CP level increased to 18%, the weight loss amount of breeding pigeons in the lactation period decreased significantly, which is consistent with previous results by Gao et al. (2016a) and Chen et al. (2016). ME level had no significant effect on the weight of the breeding pigeon, but there was a significant interaction between ME and CP on their effect on breeding pigeon weight and reproductive production. Notably, high ME in the diet but low CP levels (15–16%) caused weight loss in breeding pigeons, while high CP levels (17–18%) caused the opposite effect. This means that a very high or too low energy/protein ratio reduces the production performance of breeding pigeons. Also, high CP levels significantly shortened the average egg-laying interval during the lactation period and positively correlated with the laying rate of breeding pigeons, which is consistent with Gao et al. (2016a) and Ding et al. (2016) findings. However, too high CP levels in actual production tend to deposit too much body fat in breeders, which prolong the egg-laying interval of female breeders and even cause difficulties in the emergence of offspring, etc. (Costantini, 2010; Ding et al., 2016; Kriseldi et al., 2018). Test group 12 (18% CP, 13.0 MJ/kg), which had the lowest weight loss during lactation, did not perform as well as group 9 (17% CP, 13.0 MJ/kg) and group 10 (18% CP, 12.6 MJ/kg) in reproductive production. This study also revealed that test group 12 (18% CP, 13.0 MJ/kg), which had the lowest weight loss during lactation, did not perform as well as group 9 (17% CP, 13.0 MJ/kg) and group 10 (18% CP, 12.6 MJ/kg) in reproductive production. It has been found that changes in the composition of breeder diet formulations may affect nutrient deposition in eggs (Junqueira et al., 2006). In the present study, we found no significant effect of the interaction of CP and ME levels in breeding pigeon diets on egg quality, including mean egg weight, egg shape index, and yolk weight, among others. Similar studies have also observed in laying hens that dietary energy/protein ratios do not affect yolk weight, Haugh unit, or shell thickness (Ding et al., 2016; Heijmans et al., 2022). Therefore, an appropriate level of energy/protein ratio in the diet will be more favorable to the best weight recovery of pigeons after the lactation period, thus optimizing reproductive efficiency could achieve sustainable pigeon farming.

Given that squabs are the main source of meat in the pigeon market, the fundamental objective of the research on the energy/protein ratio requirements of lactating breeding pigeons is also to improve the survival rate and slaughter performance of squabs. Furthermore, a reasonable energy/protein ratio can also optimize the F/G to reduce production costs and maximize the output (Hu, 2016; Wang, 2018). A poor combination of protein and energy levels also affects the quality of “crop milk” from the breeding pigeon, which in turn affects the growth and development of the squabs (Gillespie et al., 2013; Fouad and El-Senousey, 2014; Gao et al., 2016b). The market weight and F/G of squabs are also the most intuitive indicators to examine the feasibility of the ration formula and the feeding effect. In this study, we found that CP and ME levels had little effect on the mortality of squabs in summer, but there was a strong interaction between ME and CP levels on their effect on the body weight and F/G of squabs. Among them, dietary ME level only influenced the feeding and body weight gain in the early growth stage of the squabs, and increasing ME level reduced feed intake. This could be due to the actual nutrient intake and feed utilization efficiency increased with an increase in the dietary nutrient concentration, which is consistent with a previous study (Sahin et al., 2003). However, we also found that the growth rate of squabs in the early growth stage showed a tendency to increase and then decrease as the ME level increased, which means that too high a level of ME in the diet can have a negative effect. Studies have shown that increasing the protein level in the diet can significantly improve feed utilization efficiency (Liu et al., 2005; Wu et al., 2005). In contrast, in the present study, we found that the optimal CP level for squabs was not the same in different growth stages. In the first and middle growth stages (1–14 d), the fastest growth rate of the squabs occurred at 16% CP level, while F/G was the lowest. In the later growth stage (14–21 d), 18% CP was optimal for weight gain and feed efficiency rate of the squabs. Based on this, we recommend that when we go on to further study the nutritional requirements of squabs, perhaps we can break down the lactation period to explore it in stages.

Slaughter performance is an important indicator of the growth performance of meat poultry, which reflects the differences in the number of nutrients deposited in different tissue parts of the diet (Chen et al., 2017). It is also an important reference indicator to evaluate the feeding management status and nutritional status of meat pigeons. These indicators clearly reflect the growth and development of squabs, while helping to better improve production efficiency. In terms of poultry slaughter performance, dietary ME levels can have a significant effect on abdominal fat deposition, while diets with higher levels of CP are more favorable for the growth of breast muscle (Cao et al., 2014). In mammals, differences in CP levels in parental diets can affect the organ or tissue quality of offspring by influencing protein or amino acid levels in milk (Wang, 2011), and a similar pattern may exist in domestic pigeons. Similar to this result, our results showed that with the change in ME and CP levels, the differences in pectoral muscle weight, abdominal fat, and organ weight between the groups of squabs were significant. High CP levels significantly improved carcass quality and the development of digestive-related organs such as the gizzard weight in squabs. High ME levels exerted the opposite effect. It is manifested in the reduction of eviscerated weight and the development of vital organs such as the heart and liver. The abdominal fat ratio is also an important indicator of lipid deposition (Zhang et al., 2022). In general, when animals consume diets with high CP levels, the bodies need to consume more energy to drive the excretion of nitrogen, which disrupts the deposition of excess fats (Wang et al., 2021). Rozenboim et al. (2016) also showed that the abdominal fat rate of broiler chickens increased with increasing dietary energy level and decreased with increasing dietary CP levels within a certain range. The present study revealed conflicting findings, in which the abdominal fat content of squabs increased with increasing CP levels. It may be necessary to explain this phenomenon further based on serum biochemical indices in squabs.

Meat physiochemical quality of squabs is also closely related to the nutritional level of the diet, and appropriate dietary energy and protein levels significantly improve the meat quality of domestic poultry (Shi et al., 2009; Lin et al., 2020). The quality of muscle is usually reflected in the meat color, pH, and drip loss. Among them, meat color is the most visual indicator of physiological and biochemical changes in muscle. Muscle brightness values are influenced by the myoglobin and fat deposition content of the muscle, with red values reflecting the myoglobin content and yellow values reflecting the influence of ration pigments (Mir et al., 2017). Many previous studies on the assessment of pectoral muscle meat color have shown that the smaller the L^⁎^ value, the larger the a^⁎^ value, and the smaller the b^⁎^ value, the better the muscle quality and vice versa (Sun, 2004; Wu et al., 2018; Wen et al., 2020). pH is an important index of muscle quality and can affect muscle color and tethering power. The lower the drip loss, the higher the turgidity and tenderness of the muscle (Zhang et al., 2015; Bernad et al., 2018). Tang et al. (2007) showed that increasing the level of ME in the diet significantly increased the b^⁎^ value and the 24-h pH of broiler pectoral muscle and decreased the drip loss rate of the muscle. In this experiment, the b^⁎^ value and the 24h pH of the pigeon pectoral muscle increased and then decreased with an increase in the ME level. This indicated that the dietary ME level should not be too high or too low, and 12.8 MJ/kg ME was the optimal ME level for the best quality pigeon meat. An increase in CP level significantly affected the 45 min meat color (L^⁎^, a^⁎^, b^⁎^) and pH (45 min, 24 h) after slaughter. Generally, a high CP level had more beneficial than harmful to the overall pectoral muscle meat color. The histological properties of muscle fiber are the histological basis of meat quality and are a major indicator for assessing meat quality. Some studies have shown that as the diameter of muscle fibers increases, muscle tenderness decreases accordingly (Zhu et al., 2020). Therefore, the finer and denser the muscle fibers are, the more tender and juicy the meat will be, and Jackson et al. (1982) further showed that diets with high ME and high CP could improve muscle fiber density. This experiment also showed that the ME and CP levels of 12.8 MJ/kg and 18%, respectively, significantly increased the muscle fiber density and reduced the cross-sectional area of pectoral muscle fibers in squabs. This demonstrates that appropriate energy/protein ratio levels greatly improve the meat quality of squabs, which ensures better slaughter performance.

Finally, a linear regression analysis was performed to estimate the energy/protein ratio requirements in the lactation diets of breeding pigeons in summer “2 + 4” breeding pattern. The correlation coefficient (*R*^2^) between each index and the energy/protein ratio level in the diets was low. This could be the effect of different energy/protein ratios was a reciprocal effect between the combined two factors, resulting in a poorer mean of the data for each treatment group. The optimal requirements for the laying rate, average laying interval, and total weight loss of breeding pigeons were all negatively correlated with the optimal requirements for actual production. For example, the model equations for the laying rate only yielded minimum values. Therefore we can only infer the optimal requirement from the range of energy/protein ratios provided in this experiment. If the F/G is the primary objective, the recommended level of energy/protein ratio in the diet of breeding pigeons is 17.92 to 19.02 kcal/g; if the reproductive efficiency is the primary objective, then the recommended energy/protein ratio in the breeder's diet is 16.72 kcal/g.

## CONCLUSIONS

The energy level in summer diet has little effect on the production of breeding pigeons during the lactation period, but the protein level significantly affects the reproductive performance of these birds. There is a strong interaction between energy and protein levels in pigeons. The best reproductive performance, the fastest growth of squabs, and better meat quality of pigeons in summer are achieved at 18% CP and 12.8 MJ/kg. Finally, the regression model revealed that the best dietary energy/protein ratio was 17.92 to 19.02 kcal/g for squabs and 16.72 kcal/g for the breeding pigeons. Therefore, 18% CP and 12.8 MJ/kg can be considered the best energy/protein ratio requirement for breeding pigeons in the lactation period under the summer “2 + 4” breeding pattern.
